# Designing concept maps for a precise and objective description of pharmaceutical innovations

**DOI:** 10.1186/1472-6947-13-10

**Published:** 2013-01-18

**Authors:** Maia Iordatii, Alain Venot, Catherine Duclos

**Affiliations:** 1 UFR SMBH, LIM&BIO, Paris 13 University 74 rue Marcel Cachin, 93017, Bobigny Cedex, France

## Abstract

**Background:**

When a new drug is launched onto the market, information about the new manufactured product is contained in its monograph and evaluation report published by national drug agencies. Health professionals need to be able to determine rapidly and easily whether the new manufactured product is potentially useful for their practice. There is therefore a need to identify the best way to group together and visualize the main items of information describing the nature and potential impact of the new drug. The objective of this study was to identify these items of information and to bring them together in a model that could serve as the standard for presenting the main features of new manufactured product.

**Methods:**

We developed a preliminary conceptual model of pharmaceutical innovations, based on the knowledge of the authors. We then refined this model, using a random sample of 40 new manufactured drugs recently approved by the national drug regulatory authorities in France and covering a broad spectrum of innovations and therapeutic areas. Finally, we used another sample of 20 new manufactured drugs to determine whether the model was sufficiently comprehensive.

**Results:**

The results of our modeling led to three sub models described as conceptual maps representingi) the medical context for use of the new drug (indications, type of effect, therapeutical arsenal for the same indications), ii) the nature of the novelty of the new drug (new molecule, new mechanism of action, new combination, new dosage, etc.), and iii) the impact of the drug in terms of efficacy, safety and ease of use, compared with other drugs with the same indications.

**Conclusions:**

Our model can help to standardize information about new drugs released onto the market. It is potentially useful to the pharmaceutical industry, medical journals, editors of drug databases and medical software, and national or international drug regulation agencies, as a means of describing the main properties of new pharmaceutical products. It could also used as a guide for the writing of comprehensive and objective texts summarizing the nature and interest of new manufactured product.

## Background

When a new drug or a new presentation of a drug is marketed, physicians need to be able to determine whether it is likely to be more useful for the treatment of their patients than the alternatives already available, and whether it is likely to modify their treatment practices. It is difficult for physicians to form their own opinions about a new drug. The pharmaceutical industry, through drug advertising, has a predominant and not always objective influence. Analyses of the official information about a new manufactured product would require considerable effort. This information is contained in different types of documents: (i) the summary of product characteristics (SPC), which forms the basis of its monograph, (ii) the SPCs of other drugs currently used for the same indication, (iii) the evaluation report for the new drug published by the national drug agencies.

Monographs have a standardized structure that varies little between countries. They include sections on composition, route of administration, indications, contraindications, adverse reactions, treatment regimens, etc. However, the nature of the novelty of the new drug is not specified clearly in the monograph, and the monograph alone provides no comparison with existing drugs for the same indications. Drug monographs provide sufficient information for the safe prescription of a given drug, but too little information to allow a physician to develop a therapeutic strategy.

When evaluating a new manufactured product with respect to existing treatments, physicians must first identify the set of drugs used for a given indication, and then study the monographs for each drug in the set, section by section, comparing the properties of each drug with those of the new drug. This process is time-consuming and unrealistic.

The national and international drug agencies (EMA [1], ANSM [2], HAS [3], FDA [4]) publish evaluation reports for each new manufactured product. These reports usually contain a comparison of the new drug with other drugs with same indications, recommendations for approval, and an overview of the clinical program, efficacy data, safety findings, recommended doses, and information for use in specific populations. These reports often conclude with a nation-specific drug-novelty index (*e.g.* in France, the Actual Benefit of the drug), which classifies the innovation as major, important or minor.

Very few physicians read these evaluation reports because they tend to be long and time-consuming to read. They also tend to focus on comparisons of the efficacy of the new manufactured product with other drugs for a given indication; aspects relating to safety or ease of use are described in less detail and may even be completely ignored. Improvements in drug labelling have been proposed, to provide patients and physicians with better information, including the results of studies comparing effectiveness [5]. Other authors [6] have highlighted problems due to a lack of information concerning the value of the innovation brought by the new drug after entry into the market. The effectiveness of drug fact boxes for communicating information about the benefits and adverse effects of drugs has been evaluated experimentally. The results obtained were encouraging, but this approach essentially targets patients and not physicians [7]. A comprehensive, but easy to read description of the various aspects of pharmaceutical innovation is required. There is therefore a need to develop new tools enabling physicians and other health professionals to comprehend the main characteristics and clinical impact of a new manufactured product both rapidly and easily. This requires the selection and structuring of the elementary pieces of information for each pharmaceutical innovation to be provided to the physician.

The objective of this study was to identify these pieces of information and to build and validate a conceptual model including the essential aspects of a new drug to be presented to physicians.

We describe the methods used for the selection and modeling of information about new drugs and provide conceptual maps defining the final model.

## Methods

### Overall approach

We constructed a preliminary conceptual model of pharmaceutical innovations based on the knowledge of two of the authors of this paper, CD and AV, who are experienced in the field of drug-information modeling [8-14]. We then refined this model, using a random sample of 40 new manufactured products that had recently been approved by the national drug authorities in France. We analyzed the French drug agency reports and SPCs of these drugs, together with those of other drugs for the same indication, with which they were to be compared. Finally, we used another sample of 20 new manufactured products from the same source to determine whether the model was sufficiently comprehensive.

### Choice of drugs used to design the model

We studied firstly the evaluation reports for 40 drugs from a list of 170 drugs that had been approved by the French National Medicines Assessment Committee between January 2008 and January 2011. These drugs are presented in Additional file 1. They were randomly selected, but taking into account the total number of new products in each medical discipline and the nature of the pharmaceutical innovation indicated in the heading of the evaluation report. In this way, we were able to obtain a sample of drugs with innovations of different natures (new molecule, new combination, new pharmaceutical form, etc.) for use in diverse medical specialties (*e.g.* cardiology, endocrinology). We excluded vaccines, which are developed by biological engineering techniques and cannot be described in the same way, and drugs for diagnostic purposes, which are used almost exclusively by radiologists.

We then use another random sample of 20 drugs from the list of 170 new drugs approved by the French drug authorities between January 2008 and January 2011. These drugs are presented in Additional file 2. They were used to investigate whether the principal innovative features of these drugs could be described with the model.

### Designing a model of pharmaceutical innovation

Step 1Two of the authors of this paper, AV and CD worked together to select an initial set of innovation axes. For each axis, they proposed a set of items corresponding to important information for a physician who wants to know whether a new drug can be useful for his daily practice.

Step 2Then author MI completed gradually the model by reading the content of (i) the evaluation reports edited by experts of Transparency Committee of the French Health Authority for each of the 40 new manufactured products, (ii) the SPCs, validated by the French Agency for the Safety of Medicine and Health Products for each new drug. Likewise she used the SPCs of the drugs to which the new drug is compared in the evaluation report for the items of the impact and the SPCs of all the drugs of the therapeutic arsenal for the items of the novelty. When she identified innovative features which could not be represented by the model, she suggested to add new items to the model. Her suggestions were then validated or not by authors AV and CD working together.

The evaluation reports contain information about pharmaceutical, toxicological, pharmacological and clinical data concerning efficacy and tolerance, including the risk/benefit ratio of the product. These reports also include information about the type of innovation (*e.g.* a new combination of drugs, a new formulation or a new dose).

The SPCs were available from drug databases [15-17]. They were used to compare various properties of the new drug with those of therapeutic arsenalpharmacological class, pharmacodynamics, contraindications, precautions, adverse reactions, dosage and administration, overdosage.

We chose to represent the information about pharmaceutical innovation with concept maps, which are used to represent knowledge models [18], particularly in the building of ontologies [19]. The graphical representation of these concept maps was generated with FreeMind software [20]. It should be stressed that, for drugs with multiple indications, a concept map must be built for each indication. For drugs for which no direct comparison studies were available, we relied on the conclusions of the experts from national drug regulation agencies.

### Analysis of the completeness of the model

The objective was to verify that all the features important to describe the innovation of a new pharmaceutical product are covered by the model.

For each of the 20 drugs of the analysis set of the completeness of the model, their evaluation reports and the SPCs were analyzed using the same methodology as in the model development. The objective was to see if the model was adequate to represent all the information related to innovation or if new items should be added.

## Results

### Three axes for describing a pharmaceutical innovation

We suggest that new pharmaceutical products should be described in terms of:

• The context of use of the new product. The medical problem for which this product is proposed should be described, together with the various existing drugs already used for this medical problem.

• The novelty of the new product. Various aspects should be considered, including chemical, pharmacological and pharmaceutical aspects.

• The impact of the new product. The impact of the new drug, in terms of its efficacy, safety and ease of use with respect to other drugs of the therapeutic arsenal should be described separately.

### Modeling the medical context of use of the new manufactured product

**Figure 1:**
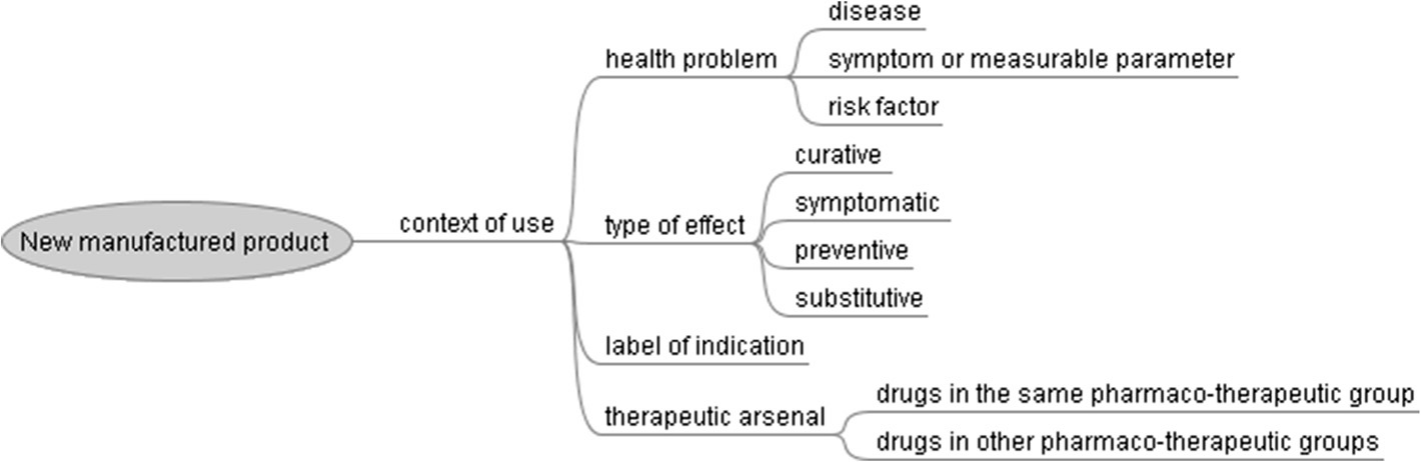
Concept map portraying the medical context of use of the new manufactured product.

The model of the medical context is presented in figure 1.

Therapeutic innovation can be defined as a new therapeutic option to treat a health problem for which there may already be an existing therapeutic arsenal. The treatment may target a disease, a symptom or a risk factor.

According to the effects of the drug, there are different types of treatment:

– Curative; the drug eliminates the cause of the disease (*e.g.* antibiotics).

– Symptomatic; the drug relieves symptoms arising from a pathological condition (*e.g.* analgesics).

– Substitutive; the drug replaces a deficient natural constituent in the organism (*e.g.* insulin for diabetics).

– Preventive; the drug is administered to healthy subjects as a preventative measure against future disease or to temporarily modify a physiological process (*e.g.* contraceptives).

Additional file 3 gives for each item of context of use, information sources where its value can be found for a given drug.

**Figure 2:**
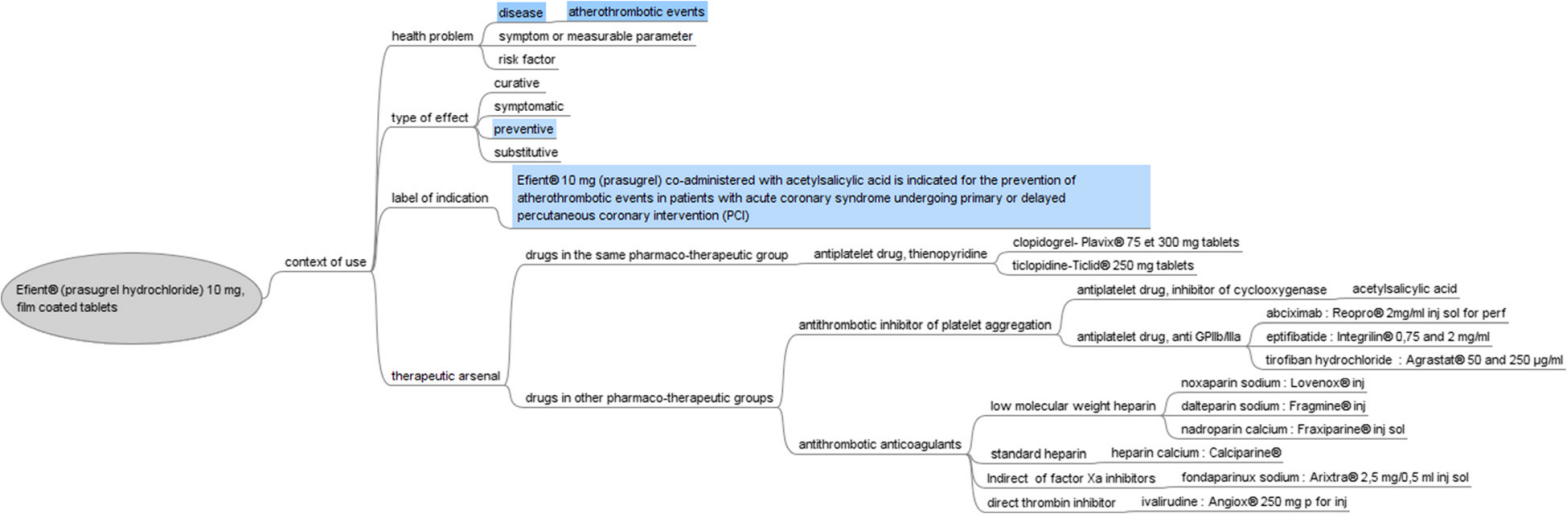
**Concept map portraying the medical context of use of Efient® (prasugrel hydrochloride 10 mg, film coated tablets) for the prevention of atherothrombotic events.** The specific elements of the new product are shown in blue.

The exact indications and conditions of use of the drug are specified on the drug label. The therapeutic arsenal for a particular condition consists of all the drugs for that indication. These drugs may or may not belong to the same pharmacotherapeutic group. Figure 2 shows the concept map built for Efient® (prasugrel hydrochloride, 10 mg, film-coated tablets). Efient® 10 mg (prasugrel) co-administered with acetylsalicylic acid is indicated for the prevention of atherothrombotic events in patients with acute coronary syndrome (i.e. unstable angina, non-ST segment elevation myocardial infarction [UA/NSTEMI] or ST segment elevation myocardial infarction [STEMI]) undergoing primary or delayed percutaneous coronary intervention (PCI).

#### Modeling the novelty of the new commercial product

**Figure 3:**
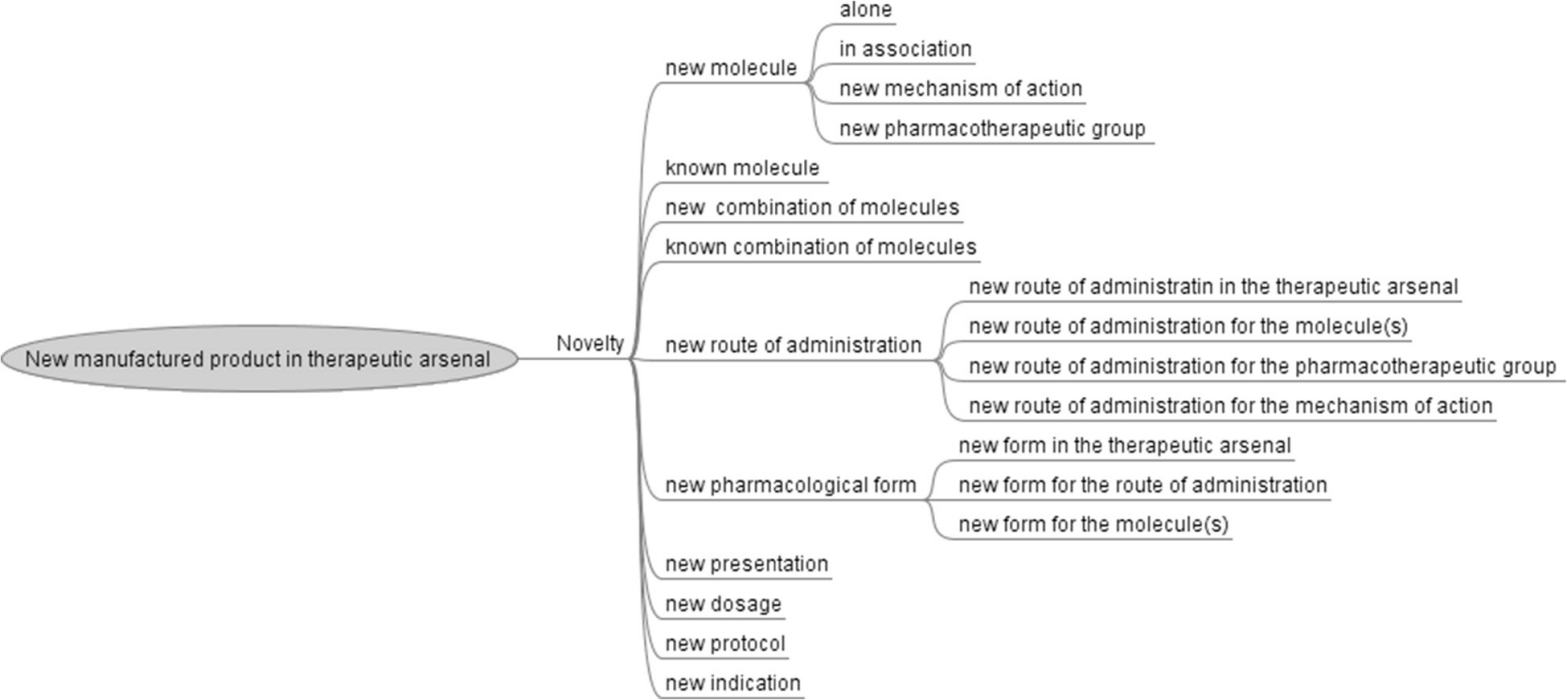
Various possible aspects of novelty for new manufactured product.

Figure 3 presents the various possible aspects of novelty for a new manufactured product. The nature of the innovation differs between drugs, which may be new molecules with or without a new mechanism of action and may belong to a new pharmacotherapeutic group. The innovative aspect may be a new combination of molecules, a known combination in a new form, a new protocol, a new dosage form, or a new presentation. The new manufactured product may incorporate only known molecules, but with new characteristics.

Additional file 4 gives for each item of novelty, information sources where its value can be found for a given drug.

The novelty may be due to a new route of administration, or a new pharmacological form, presentation, dose or protocol. The route of administration may be novel for an already existing molecule or related to the mechanism of action. For new molecules, the route of administration may be novel with respect to the whole therapeutic arsenal.

The pharmacological form may be novel for the molecule, route of administration or relative to the other drugs used for the same indication.

A new presentation of a drug may also be considered as a novelty. For example, Epipen® auto-injector, in a single dose of 0.15 mg epinephrine injection/0.3 ml sterile solution, is a drug with a new presentation of a known molecule (epinephrine). The novelty of the drug lies in the model of injector.

The 40 new drugs chosen included 11 new molecules, eight of which had a novel mechanism of action and belonged to a new pharmacotherapeutic group. Five drugs corresponded to new combinations. There were 24 drugs corresponding to known molecules, 10 of which had new features (new form, new route of administration, new presentation or new dosage) and 14 of which had a new indication or an extension of the previous indication.

**Figure 4:**
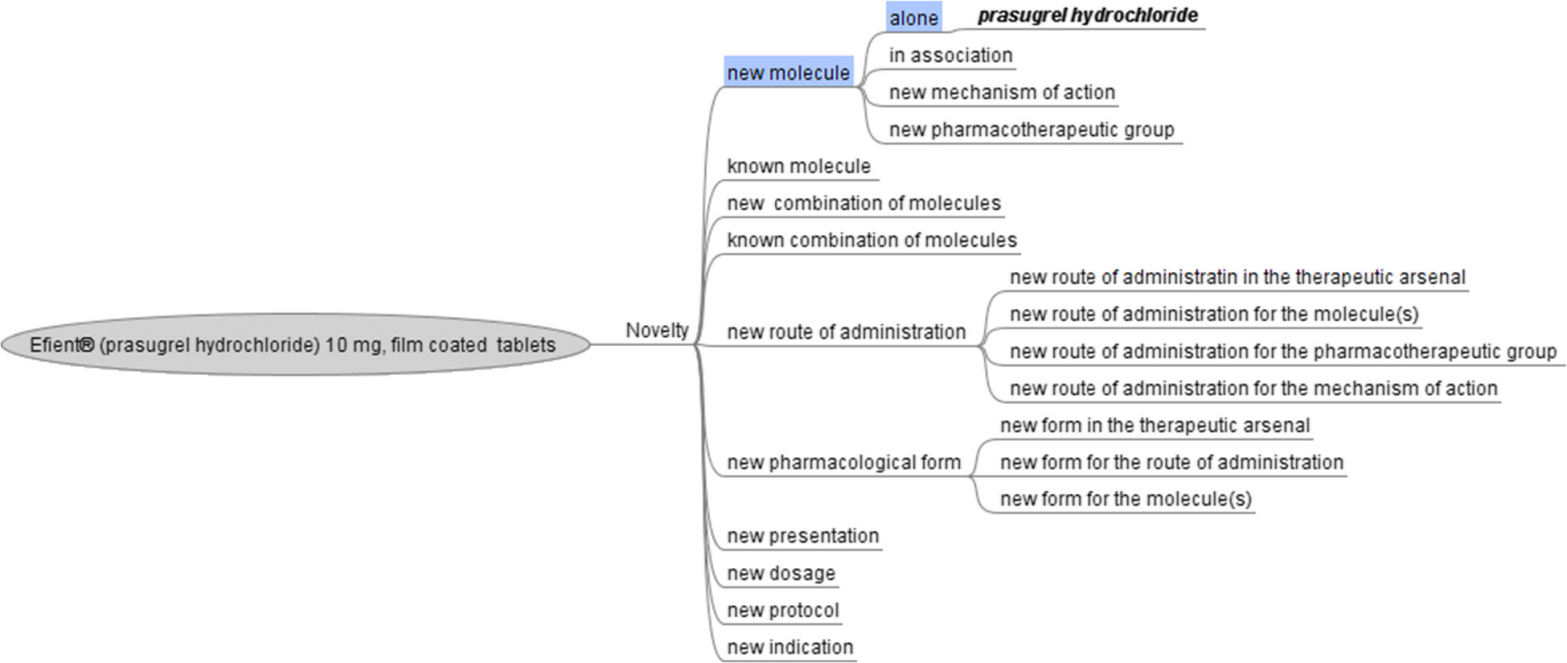
**Aspects of novelty for Efient® (prasugrel hydrochloride 10 mg, filmcoated tablets).** The specific elements of the new product are shown in blue.

An example is given in Figure 4 for the novelty of Efient®.

#### Modeling the impact of the drug

The new manufactured product must be positioned relative to the set of drug treatments sharing the same indication.

**Figure 5:**
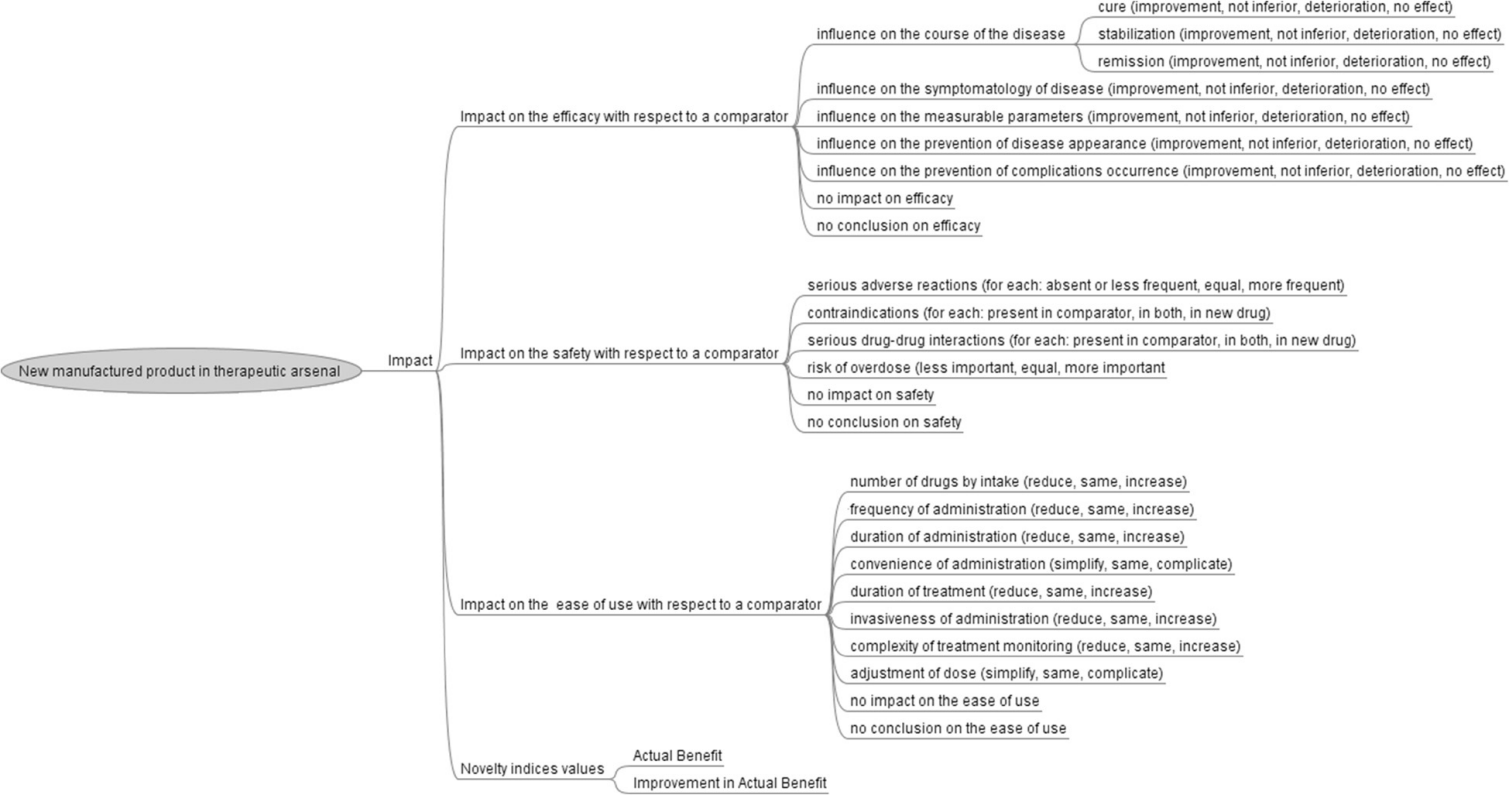
Impact of a new manufactured product with respect to a comparator.

Figure 5 shows the various elements influencing the impact of the new manufactured product with respect to a comparator. Additional file 5 gives for each item, information sources where its value can be found for a given drug.

#### Impact on efficacy

A new manufactured product may have an impact on efficacy through effects on the course of the disease (cure, stabilization or remission of the disease), on the symptomatology of the disease or on measurable parameters (*e.g.* HbA1c, systolic blood pressure), or by preventing the occurrence of the disease or its complications. The new drug may have no impact on efficacy.

#### Impact on safety

The basic characteristics of a drug likely to affect its safety include adverse reactions, contraindications, drug interactions and the risk of overdose.

We considered each of the serious adverse reactions listed in the SPC of the new product and in the ones of the comparator, determining whether or not it was present in both lists. A “serious adverse reaction” was well defined in a previous study [21] and on the website of the FDA [22].

We also considered contraindications, determining whether they were present or absent for the new product and the drugs with which it was being compared.

We also compared the serious drug-drug interactions, as defined in previous studies [23,24], listed in the SPC of the new product with those listed in the SPCs of the drugs with which it was being compared.

Another important characteristic affecting safety is the risk of drug overdose, which can be very dangerous and difficult to treat. This aspect includes the availability of an antidote.

Finally, the new product may have no impact on safety, particularly if it contains a known molecule.

#### Impact on the ease of use

The elements with a potential impact on the ease of use of drugs by patients or physicians are represented in Figure 5 and must be compared between the new product and other drugs with the same indication.

For the patient, the number of drug units, the frequency and duration of administration, the convenience of administration and its invasiveness may all influence compliance with the treatment.

For physicians, innovations may lead to treatments that are easier to monitor (*e.gcpe* enoxaparin requires more monitoring tests than rivaroxaban), doses easier to adjust.

**Figure 6:**
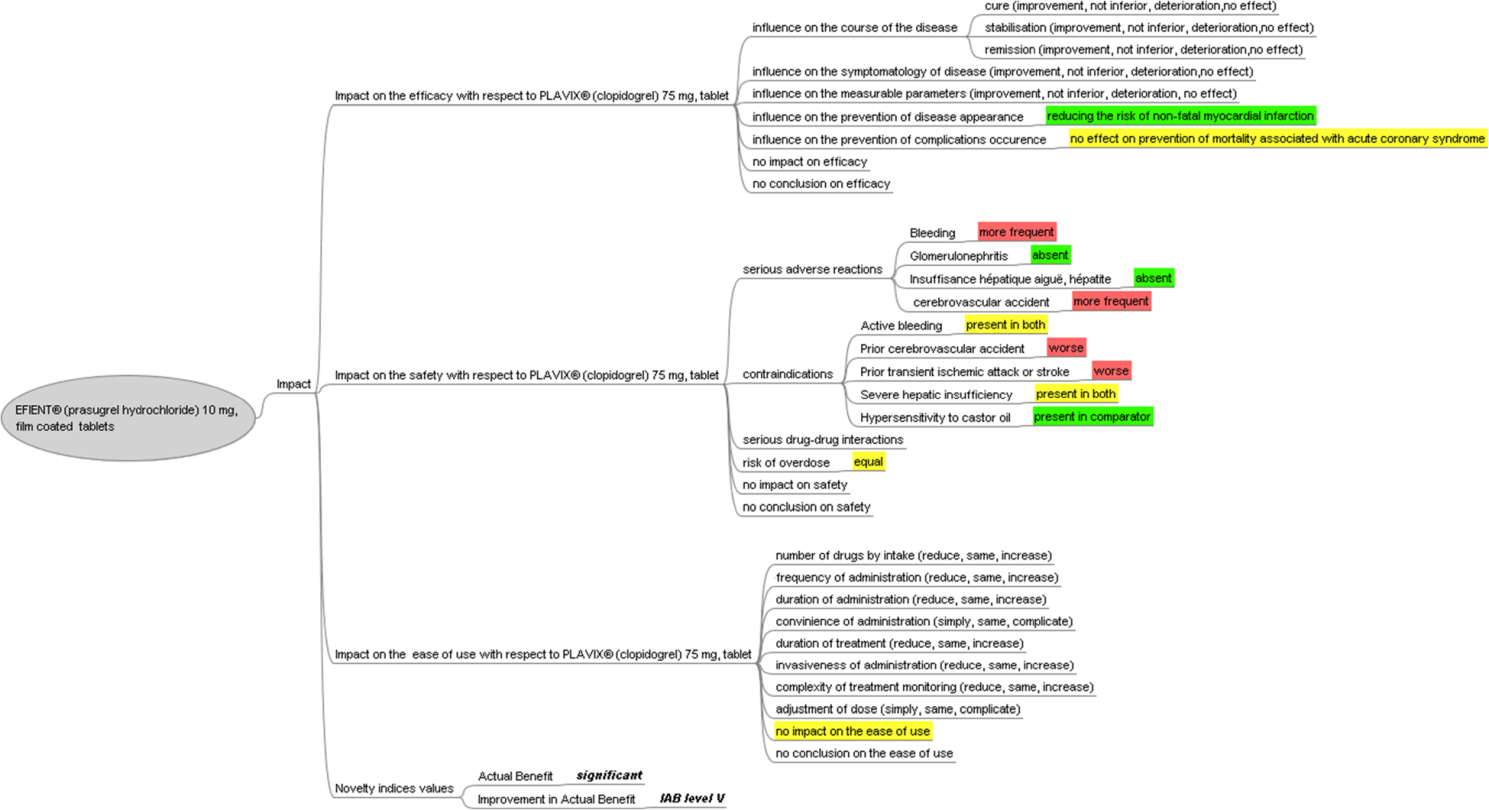
**Impact of Efient® (prasugrel hydrochloride 10 mg, film coated tablets) with respect to Plavix® (clopidogrel 75 mg, tablet).** A positive impact of the drug with respect to the comparator is shown in green; an equivalent impact is shown in yellow and a negative impact in red.

Figure 6 shows the impact of Efient® with respect to Plavix® (clopidogrel, 75 mg, tablet).

### Analysis of the completeness of the model

Six of the drugs selected for analysis of the completeness of model corresponded to new molecules; among them 5 had a new mechanism of action and belonged to a new pharmacotherapeutic group. Three drugs corresponded to new combinations and 2 to a known combination with new dosage.

Nine drugs corresponded to known moleculesthree with new features and six with a new indication. Two of the six drugs with new indications had a new mechanism of action and belonged to a new pharmacotherapeutic group for this new indication.

This analysis led to adding only one element to the model. It is the “the duration of administration” which concerns the impact on the ease of use of the treatment. This extra element was minor and did not change the overall structure of the concept maps.

## Discussion and conclusion

In this study, we identified the key items of information required for a fair appraisal of pharmaceutical innovations. The information concerning a new pharmaceutical product was separated into three categoriesi) medical context of use, including the therapeutic arsenal with same indication; ii) description of the pharmaceutical characteristics of the innovation and iii) the expected impact of the new product in terms of efficacy, safety, and ease of use for the patient and the physician, as determined by comparison with other products with the same indication.

We used concept maps to represent the various characteristics of pharmaceutical innovation. Other methods could have been used, such as the Unified Modeling Language, but concept maps have the advantage of being easy to understand for non modeling specialists [25].

Our model is based on the knowledge of two experts in the representation of drug information [8-14], as well as on the analysis of the information for 60 recently approved new drugs, covering a broad spectrum in terms of novelty and therapeutic use.

This model is therefore likely to require only slight modifications or additions for the representation of other drugs in the future.

We did not consider the cost-effectiveness of a new pharmaceutical product in our approach, because this is influenced by healthcare policy, which may differ from one country to another.

For each item we have specified the sources of information where can be found its value so that it becomes easy to build the concept maps of a new drug. But our model raises a key question. If we wish to apply this model to a new product, do we need expert opinion from specialists in the medical field corresponding to the drug indication, or can less specialized pharmacologists do the job? All the items related to the first two axes can easily be obtained, with no ambiguity, by reading the evaluation reports of national or international drug agencies and the SPCs of the new products and of the products with which they are compared. For the third axis, related to the impact of the new drug, a pharmacologist reading the same documents should be able to apply the model in most cases. However, in some cases, the conclusions of the pharmacologist concerning the impact of the new product in terms of efficacy would need to be checked by experts if the results of the clinical trials described in the evaluation reports are not sufficiently demonstrative. This point will have to be studied in the future.

It would also be of interest to submit this model to physicians for evaluation, to eventually obtain suggestions for new items that may have been omitted in this study.

Another difficulty that may be encountered in the use of this model is a lack of comparative studies for several drugs. This may make it difficult to draw firm conclusions about the impact of the drug. In such cases, the conceptual maps will need to be updated when data relating to efficacy and rare adverse reactions become available.

Our model may have various potential applications. It allows to clearly identify the questions to be addressed by someone willing to present a pharmaceutical innovation. It is therefore potentially very useful to the pharmaceutical industry as a guide for the writing of comprehensive, objective texts summarizing the nature and value of new products. It could also be used by national or international drug agencies during the evaluation of new products and could help to standardize the writing of evaluation reports for new drugs.

Medical journals and drug-database editors could use this model to summarize the main properties of new and old pharmaceutical products for prescription. Medical software editors could develop interactive user interfaces based on this model, with the aim of presenting the properties of the new pharmaceutical product to physicians. We are currently working on developing a prototype of such software for pharmaceutical innovation visualization.

## Competing interests

The authors declare that they have no competing interests.

## Authors’ contributions

MI, CD and AV carried out the literature search, defined the methods for the study and wrote the manuscript. CD and AV developed the first conceptual model of pharmaceutical innovation. MI made the compilation of the documents available for the 60 new pharmaceutical products and refined the conceptual model. MI, CD and AV wrote the initial manuscript and the revised version. All authors read and approved the final manuscript.

## Pre-publication history

The pre-publication history for this paper can be accessed here:

http://www.biomedcentral.com/1472-6947/13/10/prepub

## Supplementary Material

Additional file 1 **APPENDIX 1.** The 40 drugs used for designing the concept maps. Click here for file

Additional file 2 **APPENDIX 2.** The 20 drugs used for analysing of the completeness of the model. Click here for file

Additional file 3 **APPENDIX 3.1.** The description of Context of use with the sources of information. Click here for file

Additional file 4 **APPENDIX 3.2.** The description of the items of Novelty with the sources of information. Click here for file

Additional file 5 **APPENDIX 3.3.** The description of the items of Impact with the sources of information. Click here for file
