# Supplementary material for: Designing concept maps for a precise and objective description of pharmaceutical innovations
Source: BMC Med Inform Decis Mak. 2013 Jan 18;13:10. doi: 10.1186/1472-6947-13-10 (PMC3560234; doi:10.1186/1472-6947-13-10)
Supplement: Additional file 1 — APPENDIX 1. The 40 drugs used for designing the concept maps. [file 1472-6947-13-10-S1.doc]

APPENDIX 1: The 40 drugs used for designing the concept maps

| **Medical specialties** | **International Nonproprietary Name, Commercial Name, dosage and form** |
| --- | --- |
| **allergology** | Grass pollen - Grazax® 75,000 SQ-T, oral lyophilisate |
| **antalgy** | Fentanyl citrate - Instanyl® 50µg/dose, 100µg/dose and 200µg/dose, nasal spray solution |
|  | Fentanyl citrate - Durogesic® 12 μg/h, 25 μg/h, 50 μg/h, 75 μg/h, 100 μg/h transdermal system |
| **oncology** | Azacitidine - Vidaza® 25 mg/ml, powder for injectable suspension |
|  | Topotecan hydrochloride - Hycamtin® 0,25 mg, capsule |
|  | Doxorubicin hydrochloride - Caelyx® 2 mg/ml, solution for perfusion |
| **cardiology** | Aliskiren hemifumarate and hydrochlorothiazide – Rasilez HCT® 150 mg/12,5 mg; 150 mg/25 mg; 300 mg/12,5 mg; 300 mg/25 mg film coated tablet |
|  | Bivalirudin - Angiox® 250 mg, powder for injectable solution or for perfusion |
| **dermatology** | Ustekinumab - Stelara® 45 mg, solution for injection |
|  | Etanercept - Enbrel® 25mg/0,5ml and 50mg/1ml, single-use prefilled syringe, 25 mg/ml, powder and solvent for injectable solution, 25 mg and 50 mg, powder and solvent for injectable solution |
| **diabetology** | Sitagliptin and metformin hydrochloride - Janumet® 50 mg/850 mg and 50 mg/1 000 mg, film coated tablet |
|  | Pioglitazone hydrochloride and glimepiride - Tandemact® 30 mg/2 mg and 30 mg/4 mg, tablet |
|  | Insulin glargine - Lantus® 100 units/ml solution for injection in vial or cartridge or prefilled pens |
| **endocrinology** | Follitropin alfa - Pergoveris® 150 UI/75 UI, powder and solvent for injectable solution |
| **gastroenterology** | Esomeprazole magnesium - Inexium® 10 mg, gastro-resistant granules for oral suspension |
| **gynecology** | Ulipristal acetate - Ellaone® 30 mg, tablet |
| **hematology** | Rituximab - Mabthera® 100 mg and 500 mg, solution for injection |
| **hepatology** | Ribavirin - Rebetol® 200 mg, capsule |
|  | Peginterferon alfa-2a - Pegasys® 135 µg and 180 µg, solution for injection in prefilled injector |
| **immunology** | Human immunoglobulin - Subcuvia® 160 g/l, solution for injection |
| **infectious diseases** | Efavirenz and emtricitabine and tenofovir disoproxil fumarate - Atripla® 600 mg/200 mg/245 mg, tablets |
|  | Caspofungin acetate - Cancidas® 50 mg and 70 mg, powder for perfusion |
|  | Moxifloxacin hydrochloride - Izilox® 400 mg/250 ml, solution for perfusion |
| **insomnia** | Melatonin - Circadin® 2 mg, extended-release tablet |
| **rare diseases** | Canakinumab - Ilaris® 150 mg, powder for injectable solution |
|  | Human c1-esterase inhibitor - Berinert® 500U, powder and solvent for injectable solution / perfusion |
| **metabolism** | Tocofersolan - Vedrop® 50 mg/ml, oral solution |
| **nephrology** | Calcium acetate - Phosphosorb® 660 mg, film coated tablet |
| **neurology** | Ropinirole hydrochloride – Requip LP® 2 mg, 4 mg and 8 mg, tablet with extended release |
|  | OnabotulinumtoxinA - Botox® 50units/vial, 100 units/vial and 200 units/vial, powder for injectable solution |
| **ophthalmology** | Levocabastine hydrochloride - Levofree® 0,05 %, ophthalmic suspension in single dose container |
| **pneumology** | Montelukast sodium - Singulair® 4 mg, chewable tablet |
|  | Ambrisentan - Volibris® 5 mg and 10 mg, film coated tablet |
|  | Bosentan – Tracleer ® 62,5 mg and 125 mg, film coated tablet |
| **psychiatry** | Methylphenidate hydrochloride – Quasym LP® 10 mg, 20 mg and 30 mg, capsule with modified release |
|  | Aripiprazole - Abilify® 5 mg, 10 mg and 15 mg, tablet; 10 mg and 15 mg, orally disintegrating tablet |
| **rheumatology** | Tocilizumab - Roactemra® 20 mg/ml, concentrate solution for perfusion |
|  | Zoledronic acid - Aclasta® 5 mg, solution for perfusion |
| **emergency medicine** | Epinephrine - Epipen® 0,15 mg/0,3 ml and 0,30 mg/0,3 ml, solution for injection in prefilled injector |
| **urology** | Solifenacin succinate – Vesicare® 5 mg and 10 mg, film coated tablet |

The drugs used in model design included one drug from each of the following medical specialties: allergology, endocrinology, gastroenterology, gynecology, hematology, immunology, insomnia, metabolism, nephrology, ophthalmology, emergency medicine and urology. The drugs used for model design also included two drugs from each of the following medical specialties: antalgy, cardiology, dermatology, hepatology, rare diseases, neurology, psychiatry and rheumatology, and three drugs from each of oncology, diabetology, infectious diseases and pneumology.

Among the forty new manufactured products, fourteen were registered for new indications, eleven were new molecules, five were new combinations, five were new pharmacological forms, two were a new presentations of known drugs (*e.g*. Epipen® which includes a new autoinjector), two were from new data in our random sample of evaluation reports, and one delivered at a new dose.
