# Supplementary material for: Designing concept maps for a precise and objective description of pharmaceutical innovations
Source: BMC Med Inform Decis Mak. 2013 Jan 18;13:10. doi: 10.1186/1472-6947-13-10 (PMC3560234; doi:10.1186/1472-6947-13-10)
Supplement: Additional file 2 — APPENDIX 2. The 20 drugs used for analysing of the completeness of the model. [file 1472-6947-13-10-S2.doc]

APPENDIX 2: The 20 drugs used for analysing of the completeness of the model

| **Medical specialties** | **International Nonproprietary Name, Commercial Name, dosage and form** |
| --- | --- |
| **antalgy** | Fentanyl citrate - Effentora® 100 µg, 200 µg, 400 µg, 600 µg, and 800 µg, gingival tablet |
| **oncology** | Lapatinib - Tyverb® 250 mg, film coated tablet |
|  | Vinorelbine tartrate – Navelbine® 20 mg and 30 mg, soft capsule |
| **cardiology** | Olmesartan medoxomil and hydrochlorothiazide -Alteisduo® 40 mg/12,5mg and 40 mg/25mg, film coated tablet |
|  | Rivaroxaban - Xarelto® 10 mg, film coated tablet |
|  | Enalapril maleate and lercanidipine hydrochloride -Zanextra® 20 mg/10 mg, film coated tablet |
| **dermatology** | Calcipotriol and betamethasone dipropionate - Xamiol® 50 μg/0,5 mg/g, gel |
| **diabetology** | Sitagliptin - Januvia® 100 mg, film coated tablet |
| **endocrinology** | Cinacalcet hydrochloride - Mimpara® 30 mg, 60 mg and 90 mg, film coated tablet |
| **gastroenterology** | Methylnaltrexone bromide - Relistor® 12 mg/0,6 ml, solution for injection |
|  | Esomeprazole magnesium – Inexium® 40 mg, powder for injectable solution or for perfusion |
| **infectious diseases** | Doripenem - Doribax® 500 mg, powder for perfusion |
|  | Lopinavir and ritonavir - Kaletra® 100/25 mg, film coated tablet |
| **neurology** | Rufinamide - Inovelon® 100 mg, 200 mg, and 400 mg,film coated tablet |
|  | Levetiracetam - Keppra® 100 mg/ml, oral solution |
| **ophthalmology** | Brinzolamide and timolol – Azarga® 10 mg/ml + 5 mg/ml, collyrium in suspension |
| **pneumology** | Bosentan - Tracleer® 32 mg, dispersible tablet |
| **psychiatry** | Agomelatine - Valdoxan® 25mg, film coated tablet |
|  | Olanzapine pamoate - Zypadhera® 210 mg, 300 mg and 405 mg, powder and solvent for injectable suspension with extended release |
| **rheumatology** | Zolendronic acid monohydrate – Aclasta® 5 mg, solution for perfusion |

The 20 drugs selected for analysis of the completeness of the model included one drug from each of the following medical specialties: dermatology, diabetology, antalgy, endocrinology, ophthalmology, pneumology and rheumatology. They also included two drugs from each of the following medical specialties: oncology, gastroenterology, infectious diseases, neurology and psychiatry and three drugs from cardiology.

One of the drugs selected for study had a new dosage form, two had new forms, two had new presentations, three were new combinations, six had new indications, and six were new molecules.
