# Supplementary material for: Designing concept maps for a precise and objective description of pharmaceutical innovations
Source: BMC Med Inform Decis Mak. 2013 Jan 18;13:10. doi: 10.1186/1472-6947-13-10 (PMC3560234; doi:10.1186/1472-6947-13-10)
Supplement: Additional file 3 — APPENDIX 3.1. The description of Context of use with the sources of information. [file 1472-6947-13-10-S3.doc]

**APPENDIX 3.1: The description of Context of use with the sources of information**

| **Items of model** | | | **Source of information** |
| --- | --- | --- | --- |
| **Context of use** | Health problem | Disease | Given in the section “indications” of the SPC and evaluation report |
|  |  | Symptom or measurable parameter | Described in the section “indications” of the SPC (e.g. pain for symptom, blood pressure for measurable parameter) |
|  |  | Risk factor | Described in the section “indications” of the SPC |
|  | Type of effect | Curative | Described in the evaluation report (e.g. infectious diseases) |
|  |  | Symptomatic | Described in the evaluation report (e.g. pain) |
|  |  | Preventive | Described in the evaluation report (e.g. prevention of bleeding) |
|  |  | Substitutive | Described in the evaluation report (e.g. hormones) |
|  | Label of indication |  | Written in the SPC and evaluation report |
|  | Therapeutic arsenal | Drugs in the same pharmaco-therapeutic group | Described in the evaluation report |
|  |  | Drugs in other pharmaco-therapeutic groups | Described in the evaluation report |
