# Supplementary material for: Designing concept maps for a precise and objective description of pharmaceutical innovations
Source: BMC Med Inform Decis Mak. 2013 Jan 18;13:10. doi: 10.1186/1472-6947-13-10 (PMC3560234; doi:10.1186/1472-6947-13-10)
Supplement: Additional file 4 — APPENDIX 3.2. The description of the items of Novelty with the sources of information. [file 1472-6947-13-10-S4.doc]

**APPENDIX 3.2: The description of the items of Novelty with the sources of information**

| **Items of model** | | | **Source of information** |
| --- | --- | --- | --- |
| **Novelty** | New molecule | alone | Information deduced from the comparison to each drug belonging to the therapeutic arsenal (for the same indication) |
|  |  | in association |  |
|  |  | new mechanism on action | Information written in the section “Originality” of the evaluation report |
|  |  | new pharmacotherapeutic group | Information written in the section “Originality” of the evaluation report |
|  | Known molecule | | Information deduced from the comparison to each drug belonging to the therapeutic arsenal (for the same indication) |
|  | New combination of molecules | | Information written in the label of the evaluation report synthesis |
|  | Known combination of molecules | | Information deduced from the comparison to each drug belonging to the therapeutic arsenal (for the same indication) |
|  | New route of administration | new route of administration in the therapeutic arsenal | Information written in the label of the evaluation report synthesis |
|  |  | new route of administration for the molecule(s) | Information deduced from the comparison to each drug belonging to the therapeutic arsenal (for the same indication) |
|  |  | new route of administration for the pharmacotherapeutic group |  |
|  |  | new route of administration for the mechanism of action |  |
|  | New pharmacological form | new form in the therapeutic arsenal | Information given in the evaluation report or deduced from the comparison to each drug belonging to the therapeutic arsenal (for the same indication) |
|  |  | new form for the route of administration | Information deduced from the comparison to each drug belonging to the therapeutic arsenal (for the same indication) |
|  |  | new form for the molecule(s) | Information written in the label of the evaluation report synthesis |
|  | New presentation | | Information written in the label of the evaluation report synthesis |
|  | New dosage | | Information written in the label of the evaluation report synthesis |
|  | New protocol | | Information deduced from the comparison to each drug belonging to the therapeutic arsenal (for the same indication) |
|  | New indication | | Information written in the label of the evaluation report synthesis |
