# Supplementary material for: Designing concept maps for a precise and objective description of pharmaceutical innovations
Source: BMC Med Inform Decis Mak. 2013 Jan 18;13:10. doi: 10.1186/1472-6947-13-10 (PMC3560234; doi:10.1186/1472-6947-13-10)
Supplement: Additional file 5 — APPENDIX 3.3. The description of the items of Impact with the sources of information. [file 1472-6947-13-10-S5.doc]

APPENDIX 3.3: The description of the items of Impact with the sources of information

| **Items of model** | | | | **Source of information** |
| --- | --- | --- | --- | --- |
| **Impact** | Impact on the efficacy with respect to a comparator | Influence on the course of the disease | cure | Information described in the in clinical trials sections of the evaluation report. This information concerns the judgment criteria (endpoints). |
|  |  |  | stabilization |  |
|  |  |  | remission |  |
|  |  | Influence on the symptomatology of disease | |  |
|  |  | Influence on the measurable parameters | |  |
|  |  | Influence on the prevention of disease appearance | |  |
|  |  | Influence on the prevention of complications occurrence | |  |
|  | Impact on the safety with respect to a comparator | Serious adverse reactions | | Definition used by OMS [19] and by FDA [20]; the list of adverse reactions present in the SPC (section “Adverse reactions”) |
|  |  | Contraindications | | Absolute contraindications described in the SPC (section “Contraindications”) |
|  |  | Serious drug-drug interactions | | Definition given by [21]; the list of drug-drug interactions present in the SPC (section “Drug-drug interactions”) |
|  |  | Risk of overdose | | Information contained in the text of SPC (section “Overdosage”) |
|  | Impact on the ease of with respect to a comparator | Number of drugs by intake | | Information contained in the text of SPC (section “Dosage and administration”) |
|  |  | Frequency of administration | | Information contained in the text of SPC (section “Dosage and administration”) |
|  |  | Duration of administration | | Information contained in the text of SPC (section “Dosage and administration”). This item is specific to the infusion and to the injection in some cases. |
|  |  | Convenience of administration | | Information contained in the text of SPC (section “Dosage and administration”) |
|  |  | Duration of treatment | | Information contained in the text of SPC (section “Dosage and administration”) |
|  |  | Invasiveness of administration | | Information contained in the text of SPC (section “Dosage and administration”) |
|  |  | Complexity of treatment monitoring | | Information contained in the text of SPC (section “Warnings and precautions”) |
|  |  | Adjustment of dose | | Information contained in the text of SPC (section “Dosage and administration”) |
|  | Novelty indices values | Actual Benefit | | Information indicated in the evaluation report (may be specific to a given country) |
|  |  | Improvement in Actual Benefit | | Information indicated in the evaluation report (may be specific to a given country) |
